# Checklist of vascular plants of Klang Gates Quartz Ridge, Malaysia, a 14-km long quartz dyke

**DOI:** 10.3897/phytokeys.166.55778

**Published:** 2020-10-29

**Authors:** Ruth Kiew, Lim Chung-Lu

**Affiliations:** 1 Forest Research Institute Malaysia, 52109 Kepong, Selangor Malaysia Forest Research Institute Malaysia Kepong Malaysia

**Keywords:** Alien species, endangered species, endemic species, flora, Important Plant Area, quartz dyke, Selangor, UNESCO World Heritage Site

## Abstract

The Klang Gates Quartz Ridge (KGQR) is proposed for protection as National Heritage and as a UNESCO World Heritage Site because of its spectacular size, exceptional beauty and significant biodiversity. The checklist of vascular plants documents 314 species that comprise a unique combination that grows on lowland quartz and that is distinct from the surrounding lowland equatorial rain forest by the absence of orchids, palms, gingers and tree canopy families. The Rubiaceae, Gramineae, Moraceae, Apocynaceae, Melastomataceae and Polypodiaceae are the most speciose families. The summit vegetation at 200–400 m elevation is dominated by *Baeckea frutescens* (Myrtaceae) and *Rhodoleia championii* (Hamamelidaceae) and shows similarities to the plant community on rocky mountain peaks above 1500 m. About 11% of its species are endemic in Peninsular Malaysia and four are endemic to KGQR: *Aleisanthia rupestris* (Rubiaceae), *Codonoboea primulina* (Gesneriaceae), *Spermacoce pilulifera* (Rubiaceae), and *Ilex praetermissa* (Aquifoliaceae). All four are provisionally assessed as Critically Endangered. Two, *Eulalia milsumi* (Gramineae) and *Sonerila prostrata* (Melastomataceae), are endemic to KGQR and a few neighbouring smaller quartz dykes. They are assessed as Endangered. The KGQR is a fragile habitat and conservation management is urgently required to halt the spread of the aggressive alien grass, *Pennisetum polystachion* and to prevent further habitat degradation from visitors. Based on KGQR being a threatened habitat, its biodiverse flora, and endangered species, it qualifies as an Important Plant Area.

## Introduction

The Klang Gates Quartz Ridge (KGQR), renamed Gombak Selangor Quartz Ridge (Mohd.-Zainuddin 2015), is a 14 km-long quartz dyke (3.12N, 101.42E to 3.15N, 101.48E) 12 km northeast of Kuala Lumpur in the state of Selangor, Malaysia. Rising to about 400 m, it dominates the skyline north of Kuala Lumpur and is believed to be the longest exposed quartz dyke in the world. Running from east to west, it is flanked to the north by the Hulu Gombak Forest Reserve Extensions and the Klang Gates Dam, a large reservoir covering 207 ha that supplies water to the capital, Kuala Lumpur. To the south, it is increasingly exposed to human disturbance.

Composed of pure quartz, the dyke was exposed as the surrounding granite material weathered away revealing sheer pale grey or white vertical cliffs rising above the surrounding vegetation. Though about 200 m wide at the base, in places the summit ridge is a knife edge only a metre or so wide with precipitous drops on either side. Jagged like a dragon’s spine, it is dissected by vertical faults giving it the appearance of limestone karst, so it has been termed a pseudo-karst formation. It is pierced by three rivers that flow through narrow gullies. The pure quartz weathers to coarse sand that is very nutrient poor, has poor water retention, and has crumbled to form a steep base of colluvium with 30–35° and in places up to 60° slopes.

Adaptation to the extremely poor nutrient status and water retention has resulted in a distinctive flora with a unique assemblage of plants that includes several rare and/or endemic species restricted to the KGQR. It is markedly different from the surrounding tropical lowland rain forest not only in species composition, but also in physiognomy (with sparse stunted trees), complexity (not multi-layered and without the epiphyte flora) and lower species diversity (Saw 2010).

Its striking topography and unique flora have long attracted botanists. The first botanical collections were made by H.N. Ridley, who made three visits in 1908, 1916 and 1921 that mainly concentrated on the summit ridge flora. He wrote the first account of the flora (Ridley 1922a), describing ten new species. Henderson (1928) produced the first comprehensive listing of plants from the KGQR included in his checklist of the flowering plants of Kuala Lumpur. It was based on collections made by Forestry Department staff, in particular by Mohd Hashim in 1908, and by H.L. Hume, employed by the Federated Malay States Museum, in 1921, who discovered the new species, *Hydnocarpus humei*, that was named in his honour. Subsequently, the herbarium collection of the Federated Malay States Museum was loaned indefinitely to the Singapore Botanic Gardens Herbarium (Henderson 1928). Henderson’s listing of 265 species provided the most complete inventory of the vegetation on the steep slopes that were still forested in those days. Unfortunately, Henderson did not cite specimens. Later significant collections were made by staff of the Forest Research Institute Malaysia, principally by E.J. Strugnell in 1927 and C.F. Symington in 1933, 1935 and 1939. After a long hiatus, Kiew (1978) described a new species, *Ilex praetermissa*, she had discovered and produced the third account of the flora (Kiew 1982) based on her collections made between 1977–1982 and included for the first time a checklist of ferns collected by B. Molesworth-Allen and A.G. Piggott, who recorded *Syngramma dayi*, a fern restricted to quartz habitats.

### Effect of Human Activities on the Flora of KGQR

Being so close to the capital Kuala Lumpur, it has suffered disturbance from agricultural activities, urbanisation, visitor pressure, and the invasion of alien weeds. All these activities threaten the continued existence of its flora and species of conservation importance. On the Kuala Lumpur side, encroachment from housing and road building threatens. The north side is protected by the Hulu Gombak Forest Reserve Extensions and the Klang Gates Dam.

**Figure 1.**
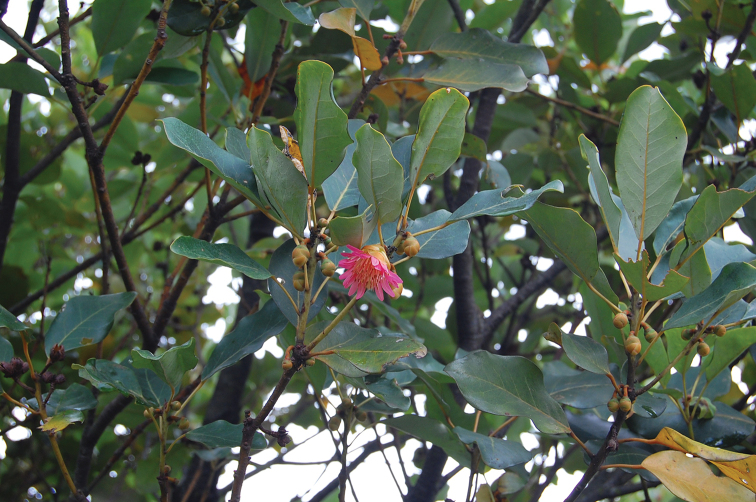
*Rhodoleia championii*, one of the dominant tree species on the summit.

Agricultural activities were a particular problem in the 1970s and 1980s (Kiew 1982; Perumal 1992) when there was widespread clearing of sections of the steep base on the south side to plant bananas and pineapple. Clearing the land by burning the vegetation got out of control and the 5 m-tall *Baeckea frutescens* trees were burned to the ground together with thick festoons up to half a metre long of the old man’s beard lichen, *Usnea* sp. Fortunately, *B. frutescens* regenerated from seed and suckers but after 30 years the old man’s beard lichen has not re-established (Kiew pers. obs.). *Rhodoleia championii* (Figure 1) survived the fires with only its leaves being scorched, but in contrast the sappy *Fagraea auriculata* was totally destroyed. Due to the nutrient-poor soil, these agricultural activities failed and the area was quickly invaded by weeds of which the most damaging was the grass *Imperata cylindrica*, a fire hazard because it becomes tinder-dry in dry weather and fuelled fires on the lower slopes.

The impact of urbanisation began with the building of a bungalow in 1883 on the top of the ridge above the gully through which the Klang River flows. Between 1893 and 1895 this gully was dammed to form a reservoir (Barlow 1995). By 1926, the bungalow had become derelict and today any sign of it has almost disappeared, although a few garden plants still persist with the patch of the native *Eriachne pallescens* grass indicating where the bungalow once stood (Kiew 1982). In the 1950s a quarry was established at the western end to utilise the quartz for glass making. It had only a very local impact and was discontinued due to lack of commercial viability. The expanding population of Kuala Lumpur required a greater water supply that resulted in enlargement of the Klang Gates Dam to its present size. This caused some local damage. Housing developments continue to creep ever closer to the KGQR. The major Kuala Lumpur-Karak Highway cuts through the western end. In 2016, a major highway development, the Eastern Klang Valley Expressway, threatened its integrity but due to public protest was re-routed away from the KGQR.

**Figure 2.**
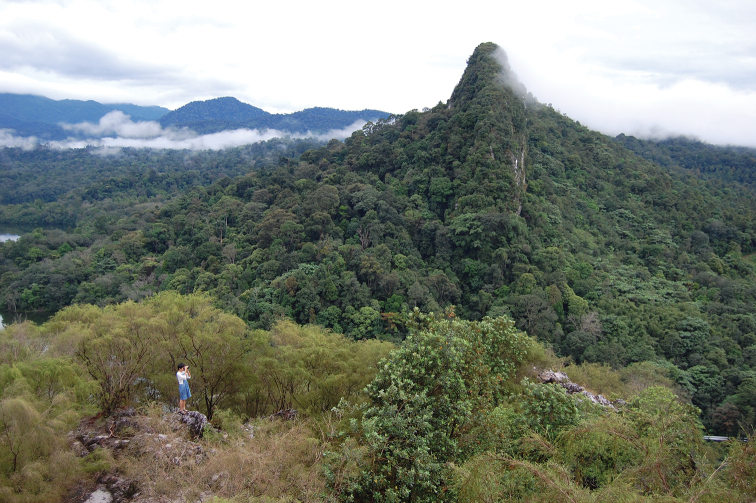
View of the eastern ridge of Klang Gates Quartz Ridge from summit of western ridge, the summit (foreground) dominated by 2–3 m tall *Baeckea frutescens*.

The KGQR’s easy accessibility and proximity to Kuala Lumpur has long encouraged rock climbers and hikers who are rewarded by a panoramic view of the Kuala Lumpur skyline in one direction and the reservoir lake and virgin rain forest in the other (Figure 2). Unfortunately, increasing visitor pressure has its negative effects including cutting down trees for camp fires (Perumal 1992). In the 1980s it was still possible to see quartz crystals 7–10 cm long, but these have long since been taken by visitors. Notable too is the disappearance from easily accessible places of *Eurycoma longifolia*, formerly a striking plant on the summit (Kiew 1982). Local Malays believe it to be a powerful aphrodisiac. The spider orchid, *Renanthera* sp., reported by Adams (1953) is also long gone. Both are the prey of opportunistic collecting by visitors. However, they may persist on inaccessible peaks.

The natural open nature of the KGQR flora makes it vulnerable to invasion by weeds. Formally, it was an island surrounded by rain forest that acted as a buffer against weed dispersal. Now this has gone from the southern side, so the KGQR is open to invasion by any weed that can withstand harsh conditions for plant growth. The agricultural activities mentioned above led to a great increase in the number of weed species recorded from the KGQR (Kiew 1982). After the initial invasion by *Imperata cylindrica*, by the 1990s Wong et al. (2010) reported that the aggressive fern *Dicranopteris linearis* covered large areas, the composite *Chromolaena odorata*, the shrub *Clidemia hirta*, the secondary forest tree *Cyrtophyllum fragrans* and a variety of grasses were already established.

### Legal Protection

In 1936, 130 ha of the KGQR were gazetted as the Klang Gates Wildlife Reserve to protect all wildlife and in particular the serow, *Capricornis sumatraensis*, a totally protected animal in Malaysia (Perumal 1992). Over the years, KGQR has been proposed to be designated as a National Nature Monument (in 1974 by the Malaysian Nature Society in the Blueprint for Conservation in Peninsular Malaysia) and to be included in the Selangor Heritage Park (Wong et al. 2010). The Hulu Gombak Forest Reserve Extensions were given enhanced protection when upgraded to Permanent Reserved Forest Status and from 2007 the KGQR lies within the Selangor State Park. In June 2015, the Selangor Town and Country Planning Department (JPBD) renamed the KGQR the Gombak Selangor Quartz Ridge and committed to protect it as National Heritage and to get it declared a UNESCO World Heritage Site on the grounds that it is a world-class geological phenomenon being the longest exposed quartz dyke in the world, as well as for its spectacular size, exceptional beauty, and its importance as a significant natural habitat for *in situ* conservation of biodiversity. It is currently on the Tentative List of World Heritage Sites.

In anticipation of the KGQR becoming a UNESCO World Heritage Site, this account aims to make available essential baseline data on the unique assemblage of plants that make up its flora by providing:

• a complete checklist of vascular plant species

• details of the endemic and rare species of conservation importance

• a complete bibliography for the botany of the KGQR.

## Materials and methods

Accessibility and proximity to Kuala Lumpur means that many botanists have from time to time collected plants there so its flora can be said to be well-collected. This has meant that it has been possible to build the checklist using herbarium specimen data from the herbaria at KEP, KLU and SING that hold the majority of KGQR collections. Herbarium codes follow *Index Herbariorum* at http://sweetgum.nybg.org/ih. Herbarium specimens provide a permanent record and, should there be questions about a species’ identity, they can be verified at any time in the future by reference to the specimen. An example of the importance of making herbarium specimens is illustrated by the case of *Hoya mappigera*, a species only described in 2011 but that had been collected from the KGQR in 1962 (*Sinclair 10730*) under the name *Hoya campanulata*.

The database software Botanical Research and Herbarium Management System (BRAHMS) in the National Herbarium of Malaysia (KEP) at the Forest Research Institute Malaysia, Selangor, Malaysia, enabled records to be extracted from its extensive holding. Not included are exotic weeds, invasive species or plants from the surrounding lowland rain forest.

For cases where species are recorded from KGQR in the literature but specimens were not cited, for instance Henderson (1928), Molesworth-Allen (1963) and Piggott (Kiew 1982), the literature source is cited in the checklist.

## Results

The checklist compiled in this study contains 314 species of vascular plants in 233 genera and 105 families (Table 1, Appendix I). The most speciose families are Rubiaceae (32 species), Gramineae (15), Moraceae (14), Apocynaceae (11), Melastomataceae (10) and Polypodiaceae (10). Genera with five or more species include: *Ficus* (11 species), *Hedyotis* (5) and *Ixora* (5). About 11% (36 species) are endemic in Peninsular Malaysia. Percentage endemism is lower than the national average of about 25% for tree species (Saw 2010). Four species are endemic to KGQR and a further two endemic to KGQR and several neighbouring smaller quartz dykes.

**Table 1.** Families, genera and species of vascular plants of Klang Gates Quartz Ridge.

Group	Families	Genera	Species
Lycophytes	1	1	2
Ferns	16	24	36
Gymnosperms	1	1	1
Flowering plants	87	207	275
**Total**	**105**	**233**	**314**

**Figure 3.**
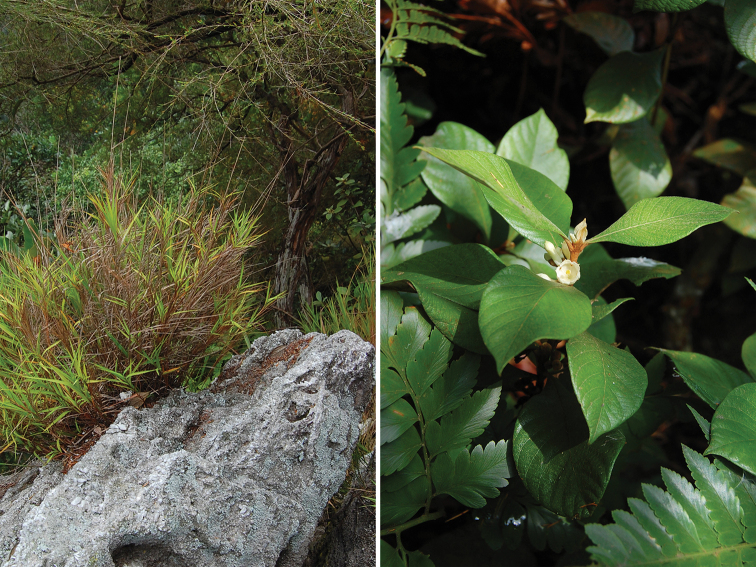
Endemic species in Klang Gates Quartz Ridge: *Eulalia milsumi* (left) and *Aleisanthia rupestris*.

In the checklist, four species proved to be endemic to KGQR, namely *Aleisanthia rupestris*, *Codonoboea primulina*, *Ilex praetermissa* and *Spermacoce pilulifera* (Figure 3). Following the IUCN criteria and categories (2001), these four species are all provisionally assessed as Critically Endangered under criteria CR B2ab(iii,iv) on the grounds that they are endemic in Peninsular Malaysia, where they are restricted to one locality that although it lies within the Selangor State Park is threatened by habitat degradation from visitor pressure and from invasive species. A further two species, *Eulalia milsumi* and *Sonerila prostrata*, endemic to KGQR and a few nearby smaller quartz dykes in the Gombak Valley, are provisionally assessed as Critically Endangered under criteria EN B2ab (iii, iv) on the grounds that they are endemic species, restricted to two to four quartz dykes that, although they lie within the Selangor State Park, are vulnerable to habitat degradation. Other species of conservation importance include *Syngramma dayi*, endemic in quartz habitats in Perak and Selangor, and a few species that are extremely rare: *Hydnocarpus humei* is known from one other collection from Larut, Perak; *Hoya mappigera* is known from one other collection from Lumut, Perak, and another from Thailand. Further, the specimen of *Galearia fulva* that Ridley described as *G. lancifolia* is strikingly different from the typical form in having extremely narrow leaves and may prove to be a distinct taxon.

## Discussion

### Habitats

Without a doubt, it is the summit flora that is of greatest botanical interest for its unique combination of species. *Baeckea frutescens* and *Rhodoleia championii* are the dominant tree species, while shrubs include *Austrobuxus nitidus* and *Vaccinium bancanum* and several epiphytic species, for instance *Fagraea auriculata*, Ficus deltoidea var. angustifolia and *Rhododendron longiflorum*, that here grow directly on the quartz rocks. The ground layer is sparse with mats of the white moss *Leucobryum aduncum* covering the thin peat layer. The endemic grass *Eulalia milsumi* forms sparse tussocks in soil-filled cracks and crannies. *Aleisanthia rupestris* as its name suggests grows in crevices on the sheer cliff faces in full sun. In contrast, *Ilex praetermissa* grows in forest on steep slopes in partial shade where there is an accumulation of peat. The gorges that pierce the ridge present a completely different cool, humid, shaded environment where lush herbs, for instance *Begonia sinuata*, *Codonoboea primulina* and gingers, can thrive.

### Comparison with other plant communities

Notable in the checklist is the absence or poor representation of trees typical of the canopy of lowland equatorial rain forest (Saw 2010), such as the Anacardiaceae, Burseraceae, Dipterocarpaceae, Guttiferae, Leguminosae, Myristicaceae, and Myrtaceae, and families like Orchidaceae, Palmae and Zingiberaceae. While it is obvious that the harsh exposed conditions and scanty soil act as a filter that excludes the majority of trees, shrubs and herbs that are typical of equatorial rain forest, it is notable that this lowland quartzite flora at 200–400 m elevation has much in common with the plant community of upper montane forest that grows above 1500 m on mountain peaks with peat that develops on weathered granitic soils (Reid 1951). In fact, Ridley (1922b) first drew attention to this phenomenon noting that KGQR included a “small but quite peculiar flora consisting of several endemic species with several only known from much higher altitudes in our mountains”. Species that illustrate this striking disjunct altitudinal distribution include *Austrobuxus nitidus*, *Baeckea frutescens*, *Dipteris conjugata*, *Oleandra neriiformis*, *Rhodoleia championii* and *Vaccinium bancanum*. Further, Whitmore (1984) drew attention to a few of these species, *Austrobuxus nitidus* and *Baeckea frutescens*, that also grow in lowland heath forest that also has base-poor, often sandy soil topped by a peat layer.

It might be expected that the quartzite flora would share similarities with the limestone flora that also grows on a rocky, free-draining substrate with poor soil development. However, comparison with the flora of Batu Caves with 269 species (Kiew 2014), a karst hill just 7 km from the KGQR, shows that in fact they share very little in common with just five species that grow in both localities, namely, *Alstonia scholaris* (a secondary forest species), *Pogonanthera pulverulenta* (an epiphyte that grows on trees, not on the rock substrate), *Microsorum membranifolium* (a lithophyte), and the figs, *Ficus hispida* and *F. punctata*. Even at the family level, the differences are very pronounced. At Batu Caves, Orchidaceae is the most speciose family with 23 species contrasting with just two orchid species on KGQR; while speciose families on KGQR, Rubiaceae (32 species), Gramineae (15 species), Melastomaceae (10 species), Polypodiaceae (10 species) and are represented by 13, 0, 1 and 2 species, respectively, on Batu Caves.

### Changes in the flora

The repeated burning of a large section of the southern face has resulted in long-term detrimental consequences. The steep slope is still covered by secondary vegetation among which the aggressive fern *Dicranopteris linearis* smothers competing vegetation. Many of the trees recorded by Henderson (1928) have not been recollected for more than 50 years, though they might still persist on the undisturbed northern side. *Sonerila prostrata* and *Spermacoce pilulifera* have not been re-collected for more than 35 years.

Among the site endemic species, *Ilex praetermissa* populations are now found only on the northern side suggesting that they are unable to disperse and become established in secondary vegetation on the southern side. This species is critically endangered having an extremely small population size and, in addition, it is a dioecious species (Kiew 1983). Wong et al. (2010) counted only 20 *Ilex* plants on a 250 m transect.

The endemic grass, *Eulalia milsumi*, is also seriously threatened by disturbance. Although reasonably common in less disturbed habitats, Wong et al. (2010) discovered that its population is highly sensitive to disturbance by aggressive smothering by weeds like *Dicranopteris linearis* and that it was significantly less frequent in disturbed areas. In addition, recently it is particularly threatened by the large, tussock forming alien grass, *Pennisetum polystachion*, first reported from KGQR by Yao (2007), but is now widespread (Kiew 2009; Lim and Yao 2010).

Fortunately, *Aleisanthia rupestris* appears to be less influenced by disturbance (Wong et al. 2010) probably because it grows in such exacting conditions in full sun on vertical rock faces rooted in tiny cracks and crevices where even weeds are unable to gain a toe-hold.

The very small population of *Codonoboea primulina* of less than 150 individuals that grows in an extremely small area measuring about 50 m^2^ (Kiew 1983), is most at risk from botanical collectors, even though there is no need for repeated collecting because it is already represented in most major herbaria in the world.

## Conclusion

The proposal by the Selangor Town and Country Planning Department to protect the KGQR under the National Heritage Act 2005 (Act 645) status is long overdue. Globally, it is indeed unique for a combination of its great size, the tall exposed quartz dyke with its pseudo-karst morphology, and its unique assemblage of plant species that includes endemic and rare plants. It meets all three criteria for being designated an Important Plant Area (Anderson 2002). The quartz dyke is a fragile, threatened habitat vulnerable to visitor pressure and invasive plant species, it harbours a unique biodiverse flora quite unlike that of either lowland forest or the limestone hill flora and of its 314 species 11% are endemic to Peninsular Malaysia, among which four are provisionally assessed as Critically Endangered and two as Endangered. Management of the dyke not only needs to control visitor access (Mohd.-Zainuddin 2015) but also to manage the invasion of aggressive weed species, most notably the *Dicranopteris linearis* thickets and to weed out *Pennisetum polystachion* by hand before it irreversibly impacts on the native flora.

## Appendix I

Checklist of vascular plant species collected from the Klang Gates Quartz Ridge, Selangor, Malaysia.

(Endemism: E – endemic in Peninsular Malaysia, EE – endemic in KGQR, E? – possibly endemic in Peninsular Malaysia).

Family classification follows the Flora of Peninsular Malaysia for ferns (Parris 2010) and flowering plants (Kiew et al. 2010).

Where details of herbarium specimens are not available, the literature source is provided, namely Henderson (1928), Molesworth-Allen (1963) and Piggott in Kiew (1982).

**LYCOPHYTES**

**
SELAGINELLACEAE
**

**^E^***Selaginella strigosa* Bedd.

Sinclair SFN 40140

*Selaginella stipulata* (Blume) Spring

Molesworth-Allen

**FERNS**

**
ADIANTACEAE
**

*Haplopteris ensiformis* (Sw.) E.H.Crane

Piggott

*Haplopteris scolopendrina* (Bory) C.Presl

Piggott

**
ASPLENIACEAE
**

*Asplenium affine* Sw.

Syahida FRI 55109

*Asplenium nitidum* Sw.

Kiew RK 1090, Parris 10968, Sinclair SFN 40142

*Asplenium pellucidum* Lam.

Molesworth-Allen, Piggott

**
BLECHNACEAE
**

*Blechnopsis orientalis* (L.) C.Presl

Piggott

**
CIBOTIACEAE
**

*Cibotium barometz* (L.) J.Sm.

Anthonysamy SA 374, Molesworth-Allen, Piggott

**
DAVALLIACEAE
**

*Davallia denticulata* (Burm.*f.*) Mett. *ex* Kuhn

Molesworth-Allen

*Davallia heterophylla* J.Sm.

Ng FRI 22112, Molesworth-Allen, Piggott

*Davallia repens* (L.*f.*) Kuhn

Piggott

*Davallia solida* (G.Forst.) Sw.

Molesworth-Allen, Piggott

**
DENNSTAEDTIACEAE
**

Microlepia speluncae (L.) T.Moore var. hancei (Ptantl) C.Chr. & Tardieu

Piggott

*Pteridium esculentum* (Forst.) Cockayne

Molesworth-Allen, Piggott

**
DIPTERIDACEAE
**

*Dipteris conjugata* Reinw.

Piggott

**
GLEICHENIACEAE
**

*Dicranopteris linearis* (Burm.*f.*) Underw.

Piggott

**
HYMENOPHYLLACEAE
**

*Hymenophyllum blandum* Racib.

Molesworth-Allen, Piggott

**
NEPHROLEPIDACEAE
**

*Nephrolepis biserrata* (Sw.) Schott

Anthonysamy SA 373

*Nephrolepis falciformis* J.Sm.

Piggott

**
OLEANDRACEAE
**

*Oleandra neriiformis* Cav.

Kiew RK 1080, Piggott

**
POLYPODIACEAE
**

*Drynaria rigidula* (Sw.) Bedd.

Piggott

*Goniophlebium percussum* (Cav.) W.H.Wagner & Grether

Piggott

*Lepisorus longifolius* (Blume) Holttum

Strugnell FMS 14623

*Leptochilus macrophyllus* (Blume) Noot.

Piggott

*Microsorum membranifolium* (R.Br.) Ching

Ingram FMS 14605

*Pyrrosia angustata* (Sw.) Ching

Symington 39403, Piggott

*Pyrrosia lanceolata* (L.) Farw.

Anthonysamy SA 372, Piggott

*Selliguea heterocarpa* (Blume) Blume

Piggott

*Selliguea lateritia* (Baker) Hovenkamp

Kiew RK 1088

*Selliguea stenophylla* (Blume) Parris

Piggott

**
PTERIDACEAE
**

*Pteris biaurita* L.

Strugnell FMS 14603

*Pteris longipinnula* Wall. *ex* J.Agardh

Molesworth-Allen, Piggott

**^E^***Syngramma dayi* (Bedd.) Bedd.

Parris 10967, Sinclair SFN 40139

**
SINOPTERIDACEAE
**

*Cheilanthes tenuifolia* (Burm.*f.*) Sw.

Piggott

**
TECTARIACEAE
**

*Tectaria fissa* (Kunze) Holttum

Kiew RK 1089, 1091, Parris 10969, Hume 7065

*Tectaria singaporiana* (Wall. *ex.* Hook. & Grev.) Copel.

Molesworth-Allen

**
WOODSIACEAE
**

*Diplazium polypodioides* Blume

Ingram FMS 14602

**GYMNOSPERMS**

**
GNETACEAE
**

Gnetum gnemon L. var. brunonianum (Griff.) Markgr.

Henderson

*Gnetum* sp.

Kiew RK 991

**Flowering Plants**

**
ACANTHACEAE
**

Peristrophe acuminata Nees var. acuminata

Henderson

*Pseuderanthemum graciliflorum* Ridl.

Henderson

**^E^***Pseuderanthemum selangorense* (C.B.Clarke) Ridl.

Henderson

*Staurogyne kingiana C.B.Clarke*

Henderson

**
ACHARIACEAE
**

**^E^***Hydnocarpus humei* Ridl.

Hume 7256

**^E^***Ryparosa fasciculata* King

Henderson

**
ANACARDIACEAE
**

*Buchanania sessilifolia* Blume

Henderson

*Semecarpus velutina* King

Mohd. Hashim FMS 102

*Swintonia schwenckii* Teijsm. & Binn. *ex* Hook.*f*.

Henderson

**
ANISOPHYLLEACEAE
**

*Anisophyllea corneri* Ding Hou

Watson 538, Kiew RK 992

**
ANNONACEAE
**

*Alphonsea elliptica* Hook.*f.* & Thomson

Syahida FRI 55106

*Mitrella kentii* (Blume) Miq.

Symington KEP 39393

*Trivalvaria pumila* (King) J.Sinclair

Sinclair SFN 40143, Kiew RK 1170

**
APOCYNACEAE
**

*Alstonia scholaris* (L.) R.Br.

Henderson

*Chilocarpus costatus* Miq.

Henderson

*Dischidia bengalensis* Colebr.

Kiew RK 211

*Epigynum ridleyi* King & Gamble

Henderson

**^E^***Hoya campanulata* Blume

Strugnell FMS 13033

*Hoya mappigera* Rodda & Simonsson

Sinclair SFN 10730

*Hoya revoluta* Wight

Kiew RK 1155, Strugnell FMS 55109

*Kibatalia maingayi* (Hook.*f.*) Woodson

Watson 533

*Pottsia laxiflora* (Blume) Kuntze

Henderson

*Willughbeia edulis* Roxb.

Mohd. Hashim FMS 402, Kiew FRI 65534, Symington KEP 39395

**
AQUIFOLIACEAE
**

**^EE^***Ilex praetermissa* Kiew

Kiew RK 215, RK 1255, Strugnell FMS 33213, Symington KEP 39398

**
ARACEAE
**

*Aglaonema nebulosum* N.E.Br.

Henderson

*Amydrium medium* (Zoll. & Moritizi) Nicolson

Henderson

*Anadendrum microstachyum* (de Vriese & Miq.) Backer & Alderw.

Kiew RK 1150

*Homalomena humilis* (Jack) Hook.*f*.

Hay AH 2023, Kiew RK 1175, Saw FRI 34265

*Homalomena pendula* (Blume) Bakh.*f*.

Hay AH 2024

*Rhaphidophora montana* (Blume) Schott

Henderson

*Schismatoglottis scortechinii* Hook.*f*.

Henderson

**
ARALIACEAE
**

*Arthrophyllum diversifolium* Blume

Henderson

*Schefflera oxyphylla* (Miq.) R.Vig.

Henderson

**
ARISTOLOCHIACEAE
**

*Thottea piperiformis* (Griff.) Mabb.

Kiew RK 1139, Stone 15648

**
BEGONIACEAE
**

**^E^***Begonia holttumii* Irmsch.

Henderson 7291

*Begonia wrayi* Hemsl.

Ridley 13430

*Begonia sinuata* Wall. *ex* Meisn.

Kiew RK 1084, Sinclair SFN 40132, Saw FRI 34264

**
CAMPANULACEAE
**

*Lobelia zeylanica* L.

Henderson

**
CAPPARACEAE
**

*Capparis versicolor* Griff.

Ridley *s.n.*, Henderson

**
COMMELINACEAE
**

*Amischotolype gracilis* (Ridl.) I.M.Turner

Ridley s.n. (1908), Kiew RK 1180

**
COMPOSITAE
**

*Blumea balsamifera* (L.) DC.

Henderson

*Vernonia arborea* Buch.-Ham.

Henderson

**
CONNARACEAE
**

**^E^***Rourea rugosa* Planch.

Henderson

**
CONVOLVULACEAE
**

*Argyreia capitiformis* (Poir.) Ooststr.

Henderson

*Neuropeltis maingayi* Peter *ex* Ooststr. var. maingayi

Henderson

**
COSTACEAE
**

*Cheilocostus speciosus* (J.Koenig) C.D.Specht.

Henderson

**
CYPERACEAE
**

*Cyperus compressus* L.

Henderson

*Cyperus cyperoides* (L.) Kuntze

Kiew RK 1142

*Cyperus laxus* Lam.

Kiew RK 1147A

*Fimbristylis thouarsii* (Kunth) Merr.

Symington KEP 33210, KEP 39410

*Mapania palustris* (Hassk. *ex* Steud.) Fern-Vill.

Kiew RK 1144

**
DICHAPETALACEAE
**

**^E^***Dichapetalum griffithii* (Hook.*f.*) Engl.

Henderson

**
DILLENIACEAE
**

*Tetracera asiatica* (Lour.) Hoogl.

Julius FRI 54873

**
DIOSCOREACEAE
**

*Dioscorea pyrifolia* Kunth

Anthonysamy SA 195

**
DIPTEROCARPACEAE
**

*Shorea bracteolata* Dyer

Watson 741

Shorea parvifolia Dyer subsp. parvifolia

Watson 739

**
DRACAENACEAE
**

*Dracaena elliptica* Thunb.

Kiew RK 224

**^E?^***Dracaena maingayi* Hook.*f*.

Kiew RK 1124, RK 1140

*Dracaena umbratica* Ridl.

Kiew RK 1194

**
EBENACEAE
**

*Diospyros sumatrana* Miq.

Henderson

**
ELAEOCARPACEAE
**

*Elaeocarpus mastersii* King

Kiew RK 77, Symington 33207, Burkill SFN 10028

Elaeocarpus nitidus Jack var. nitidus

Phoon 108, 110

Elaeocarpus stipularis Blume var. stipularis

Phoon 107

**
ERICACEAE
**

*Rhododendron longiflorum* Lindl.

Ridley s.n., Symington KEP 33215

Vaccinium bancanum Miq. var. tenuinervium J.J.Sm.

Symington KEP 33206, 39404

**
EUPHORBIACEAE
**

*Croton oblongus* Burm.*f*.

Henderson

*Epiprinus malayanus* Griff.

Henderson

*Euphorbia ridleyi* Croizat.

Wong FRI 35270

*Macaranga gigantea* (Rchb.*f.* & Zoll.) Müll.Arg.

Henderson

*Macaranga hullettii* King *ex* Hook.

Henderson

*Macaranga hypoleuca* (Rchb.*f.* & Zoll.) Müll.Arg.

Henderson.

*Mallotus macrostachyus* (Miq.) Müll.Arg.

Henderson

*Pimelodendron griffithianum* (Müll.Arg.) Benth.

Henderson

**
FAGACEAE
**

*Castanopsis inermis* (Lindl. *ex* Wall.) Benth. & Hook.*f*.

Mohd Hashim FMS 305

*Castanopsis megacarpa* Gamble

Henderson

*Lithocarpus ewychkii* (Korth.) Rehder

Henderson

*Lithocarpus sundaicus* (Blume) Rehder

Mohd Hashim FMS 29

**
GENTIANACEAE
**

*Fagraea auriculata* Jack

Henderson

**
GESNERIACEAE
**

*Aeschynanthus pulcher* (Blume) G.Don

Kiew RK 1081, Syahida FRI 55108

**^EE^***Codonoboea primulina* (Ridl.) Kiew

Ridley s.n., Kiew RK 1182, Soh FRI 471218, Syahida FRI 55105

*Codonoboea quinquevulnera* (Ridl.) C.L.Lim

Kiew RK 1077, Symington KEP 39407, Syahida FRI 55107

**
GRAMINEAE
**

*Acroceras tonkinense* (Balansa) C.E.Hubb.

Chew FRI 51872

*Axonopus compressus* (Sw.) P.Beauv.

Chew FRI 51866

*Centotheca lappacea* (L.) Desv.

Chew FRI 51873

*Chrysopogon aciculatus* (Retz.) Trin.

Chew FRI 51868

*Cyrtococcum patens* (L.) A.Camus

Chew FRI 51875

*Digitaria fuscescens* (J.Presl) Henrard

Chew FRI 51864

*Eragrostis brownii* (Kunth) Nees

Chew FRI 51863

*Eriachne pallescens* R.Br.

Chew FRI 51862, Kiew RK 1126, Symington KEP 47125

**^EE^***Eulalia milsumi* Ridl.

Symington KEP 39405, Chew FRI 51879

*Lophatherum gracile* Brongn.

Chew FRI 51871

*Melinis repens* (Willd.) Zizka

Chew FRI 51867

*Ottochloa nodosa* (Kunth) Dandy

Chew FRI 51874

*Panicum brevifolium* L.

Chew FRI 51876

*Paspalum conjugatum* P.J.Bergius

Chew FRI 51880

*Pennisetum polystachion* (L.) Schult.

Chew FRI51865

**
GUTTIFERAE
**

**^E^**Calophyllum ferrugineum Ridl. var. oblongifolium (T.Anderson) P.F.Stevens

Wyatt-Smith 66609

**^E^***Mesua elegans* (King) Kosterm.

Ridley 13527

**^E^** Mesua kunstleri (King) Kosterm. var. kunstleri

Symington KEP 47132

**
HAMAMELIDACEAE
**

*Rhodoleia championii* Hook.*f*.

Foxworthy KEP 10031, Strugnell KEP 10991, Kiew RK 210

**
HUGONIACEAE
**

*Indorouchera griffithiana* Planch. Hallier *f*.

Henderson

**
ICACINACEAE
**

*Gomphandra quadrifida* (Blume) Sleumer

Henderson

**
IXONANTHACEAE
**

*Ixonanthes icosandra* Jack

Henderson

*Ixonanthes reticulata* Jack

Symington KEP 33220, 37450

**
LABIATAE
**

*Callicarpa longifolia* Lam.

Henderson

*Callicarpa pentandra* Roxb.

Henderson

*Clerodendrum deflexum* Wall.

Henderson

*Rotheca serrata* (L.) Steane & Mabb.

Kiew RK 209, Anthonsamy SA 163

**^E^***Vitex longisepala* King & Gamble

Henderson

**
LAURACEAE
**

*Alseodaphne nigrescens* (Gamble) Kostem.

Henderson

*Litsea umbellata* (Lour.) Merr.

Mohd Hashim FMS 198

*Litsea costalis* (Nees) Kosterm.

Mohd Hashim FMS 277

**
LECYTHIDACEAE
**

*Barringtonia macrostachya* (Jack) Kurz

Kiew RK 1107

*Barringtonia scortechinii* King

Kiew RK 1179

**
LEGUMINOSAE
**

*Archidendron contortum* (Mart.) I.C.Nielsen

Henderson

*Archidendron jiringa* (Jack) I.C. Nielsen

Henderson

*Derris elegans* Grah. *ex* Benth.

Strugnell FMS13389

*Bauhinia bidentata* Jack

Mead FMS 30765

*Flemingia strobilifera* (L.) W.T.Aiton

Strugnell FMS 13387

**^E^***Fordia albiflora* (Prain) Dasuki & Schot

Henderson

**
LOGANIACEAE
**

*Norrisia malaccensis* Gardner

Symington KEP 37448

**
LORANTHACEAE
**

*Macrosolen cochinchinensis* (Lour.) Tiegh.

Symington KEP 37446, Stone 15641

*Scurrula ferruginea* (Jack) Danser

Kiew RK 1125

**
LOWIACEAE
**

**^E^***Orchidantha longiflora* (Scort.) Ridl.

Henderson

**
MALVACEAE
**

*Durio griffithii* (Mast.) Bakh.

Symington KEP 47126

*Grewia laevigata* Vahl

Henderson

*Microcos tomentosa* Sm.

Henderson

*Pterospermum javanicum* Jungh.

Watson 531

**
MARANTACEAE
**

*Donax canniformis* (G.Forst.) K.Schum.

Henderson

**
MELASTOMATACEAE
**

**^E^***Anerincleistus pauciflorus* Ridl.

Sinclair SFN 40137

*Diplectria divaricata* Kuntze

Henderson

*Medinilla crassifolia* (Reinw. *ex* Blume) Blume

Poore 1086

*Melastoma malabathricum* L.

Omar FMS 9936

*Oxyspora bullata* (Griff.) J.F.Maxwell

Kiew RK 1178

*Oxyspora exigua* (Jack) J.F.Maxwell

Henderson

*Pogonanthera pulverulenta* (Jack) Blume

Kiew RK 993, Putz FRI 21901, Wyatt-Smith KEP 66610

*Pternandra echinata* Jack

Henderson

*Sonerila obliqua* Korth.

Kiew RK 1086, Sinclair SFN 40133

**^E^***Sonerila prostrata* Ridl.

Ridley s.n., Foxworthy KEP 10039, Symington KEP 47134

**
MELIACEAE
**

Chisocheton pentandrus (Blanco) Merr. subsp. paucijugus (Miq.) Mabb.

Henderson

*Dysoxylum arborescens* (Blume) Miq.

Mohd Hashim FMS 1310

**
MELIOSMACEAE
**

*Meliosma sumatrana* (Jack) Walp.

Henderson

**
MEMECYLACEAE
**

Memecylon dichotomum (C.B.Clarke) King var. dichotomum

Henderson

**
MENISPERMACEAE
**

*Pericampylus glaucus* (Lam.) Merr.

Henderson

**
MORACEAE
**

*Artocarpus gomezianus* Wall. *ex* Trécul

Henderson

*Ficus chartacea* (Wall. *ex* Kurz) King

Henderson

Ficus deltoidea Jack var. angustifolia (Miq.) Corner

Mohd Hashim FMS 1103, Kiew RK 214, Strugnell FMS 13029

Ficus deltoidea Jack var. kunstleri (King) Corner

Kiew RK 93, Mead FMS 30761, Symington KEP 39390

*Ficus fulva* Reinw. *ex* Blume

Henderson

*Ficus hispida* L.*f*.

Anthonysamy SA 167

Ficus obscura Blume var. borneensis (Miq.) Corner

Henderson

*Ficus pellucidopunctata* Griff.

Henderson

*Ficus punctata* Thunb.

Davies 2837

*Ficus sagittata* Vahl

Henderson

*Ficus sumatrana* Miq.

Symington KEP 39394

*Ficus trichocarpa* Blume

Henderson

Ficus villosa Blume var. villosa

Henderson

*Hullettia dumosa* King

Kiew RK 1171

**
MYRISTICACEAE
**

*Gymnacranthera forbesii* (King) Warb.

Henderson

*Horsfieldia majuscula* (King) Warb.

Henderson

*Horsfieldia polyspherula* (Hook.*f. ex* King) J.Sinclair var. sumatrana (Miq.) W.J.de Wilde

Watson FMS 537

*Knema furfuracea* (Hook.*f.* & Thomson) Warb.

Henderson

*Knema hookeriana* (Wall. *ex* Hook.*f.* & Thomson) Warb.

Henderson

*Knema malayana* Warb.

Henderson

**^E^***Knema plumulosa* J.Sinclair

Mohd Hashim FMS 279

*Myristica cinnamomea* King

Kiew RK 222

**
MYRSINACEAE
**

**^E^***Antistrophe caudata* King & Gamble

Kiew RK 1181, Sinclair SFN 40132

*Ardisia colorata* Roxb.

Henderson

*Ardisia lanceolata* Roxb.

Henderson

*Ardisia villosa* Roxb.

Henderson

*Grenacheria amentacea* Mez

Henderson

*Grenacheria lampani* Mez

Henderson

*Labisia pumila* (Blume) Fern.-Vill.

Kiew RK 1148

**
MYRTACEAE
**

*Baeckea frutescens* L.

Symington KEP 37441

*Syzygium attenuatum* (Miq.) Merr. & L.M.Perry

Strugnell FMS 13036, Symington KEP 33202

*Syzygium chloranthum* (Duthie) Merr. & L.M.Perry

Mead FMS 30764

*Syzygium gratum* (Wight) S.N.Mitra

Kiew RK 850

*Syzygium subdecussatum* (Wall. *ex* Duthie) I.M.Turner var. subdecussatum

Kiew RK 213, Symington KEP 47123

**
OCHNACEAE
**

*Campylospermum serratum* (Gaertn.) Bittrich & M.C.E.Amaral

Henderson

**
OLEACEAE
**

*Jasminum elongatum* (P.J.Bergius) Willd.

Kiew RK 1154

**
OPILIACEAE
**

*Champereia manillana* (Blume) Merr.

Henderson

*Lepionurus sylvestris* Blume

Henderson

**
ORCHIDACEAE
**

*Renanthera* sp. (Spider orchid)

Adam obs.

*Dendrobium acerosum* Lindl.

Strugnell FMS 13398

**
PALMAE
**

*Calamus javensis* Blume

Henderson

*Eugeissona tristis* Griff.

Adams obs, Kiew obs.

*Licuala triphylla* Griff.

Kiew RK 1079, RK 1172

*Pinanga disticha* (Roxb.) Blume *ex* H.Wendl.

Kiew RK 1173

**
PANDACEAE
**

*Galearia fulva* (Tul.) Miq.

Hume 7146

**
PANDANACEAE
**

*Benstonea ornata* (Solms.) Callm. & Buerki

Kiew RK 1145, Rk 1810

**
PENTAPHRAGMATACEAE
**

*Pentaphragma horsfieldii* (Miq.) Airy Shaw

Henderson

**
PENTAPHYLACACEAE
**

*Eurya acuminata* DC.

Henderson

**
PHRYMACEAE
**

*Cyrtandromoea grandis* Ridl.

Kiew RK 1146

**
PHYLLANTHACEAE
**

*Antidesma salicinum* Ridl.

Henderson

*Aporosa benthamiana* Hook.*f*.

Henderson

*Baccaurea brevipes* Hook.*f*.

Henderson

*Breynia discigera* Müll.Arg.

Henderson

*Bridelia tomentosa* Blume

Burkill SFN 10033, Julius FRI 54866

*Glochidion superbum* Baill.

Henderson

*Phyllanthus pulcher* Wall.

Henderson

*Sauropus androgynus* (L.) Merr.

Henderson

**
PICRODENDRACEAE
**

*Austrobuxus nitidus* Miq.

Kiew RK 848, Symington KEP 37444, Wyatt-Smith KEP 6612

**
PIPERACEAE
**

**^E?^***Piper porphyrophyllum* N.E.Br.

Henderson

*Piper stylosum* Miq.

Kiew RK 1176

**
POLYGALACEAE
**

*Salomonia cantoniensis* Lour.

Kiew RK 208

*Xanthophyllum griffithii* Hook.*f. ex* A.W.Benn.

Watson 529

*Xanthophyllum wrayi* King

Henderson

**
PRIMULACEAE
**

*Maesa ramentacea* (Roxb.) A.DC.

Henderson

**
PROTEACEAE
**

*Helicia attenuata* (Jack) Blume

Hume FMS 7251

**
RHIZOPHORACEAE
**

*Carallia eugenoidea* King

Ridley s.n. (1921), Strugnell FMS 33208, Symington KEP 39396

*Carallia suffruticosa* Ridl.

Sinclair SFN 40136

**
ROSACEAE
**

Prunus grisea (Blume) Kalkman var. tomentosa

Henderson

**
RUBIACEAE
**

**^EE^***Aleisanthia rupestris* (Ridl.) Ridl.

Ridley s.n., Symington KEP 33201, Kiew RK 216

*Argostemma pictum* Wall.

Kiew RK 1185

*Chassalia curviflora* (Wall.) Thwaites

Henderson

*Greenea corymbosa* (Jack) K.Schum.

Kiew RK 1153

*Hedyotis auricularia* L.

Henderson

*Hedyotis capitellata* Wall. *ex* G.Don

Henderson

*Hedyotis corymbosa* (L.) Lam.

Henderson

*Hedyotis dichotoma* J.Koenig *ex* Roth

Kiew RK 207

*Hedyotis vestita* R.Br. *ex* G.Don

Henderson

*Ixora concinna* R.Br. *ex* Hook.*f*.

Kiew RK 1143

*Ixora congesta* Roxb.

Kiew RK 1152

Ixora javanica (Blume) DC. var. javanica

Henderson

*Ixora lobbii* Loudon

Henderson

Ixora pendula Jack var. pendula

Kiew RK 1151

*Lasianthus densifolius* Miq.

Henderson

*Lasianthus maingayi* Hook.*f*.

Henderson

**^EE^***Lasianthus oblongus* King & Gamble

Henderson

*Mitragyna speciosa* (Korth.) Havil.

Henderson

*Mussaenda villosa* Wall. *ex* G.Don

Henderson

*Nauclea subdita* (Korth.) Steud.

Henderson

*Neonauclea pallida* (Reinwa. *ex* Havil.) Bakh.*f*.

Henderson

*Ophiorrhiza communis* Ridl.

Henderson

*Ophiorrhiza discolor* R.Br.

Henderson

*Pavetta graciliflora* Wall. *ex* Ridl.

Henderson

*Porterandia anisophylla* (Jack *ex* Roxb.) Ridl.

Henderson

**^E?^***Psychotria maingayi* Hook.*f*.

Henderson

*Psydrax* sp.

Kiew RK 213

*Rothmannia macrophylla* (R.Br.) Bremek.

Kiew RK 1149

**^EE^***Spermacoce pilulifera* (Ridl.) I.M.Turner

Henderson

*Timonius wallichianus* (Korth.) Valeton

Kiew RK 989

Uncaria lanosa Wall. var. glabra (Blume) Ridsdale

Henderson

*Urophyllum hirstum* (Wight) Hook.*f*.

Henderson

**
RUTACEAE
**

Glycosmis chlorosperma Spreng. var. chlorosperma

Henderson

**
SALICACEAE
**

*Homalium caryophyllaceum* Benth.

Henderson

*Osmelia maingayi* King

Henderson

**
SAPINDACEAE
**

*Guioa diplopetala* (Hassk.) Radlk.

Symington KEP 37447

*Lepisanthes tetraphylla* Radlk.

Kiew RK 1141, Symington Keo 37449

*Pometia pinnata* J.Forst. & G.Forst.

Henderson

**
SAPOTACEAE
**

*Payena lucida* DC.

Henderson, Mohd Hashim FMS 37449

**
SIMAROUBACEAE
**

*Eurycoma longifolia* Jack

Kiew RK 990, RK 1073

**
SMILACACEAE
**

*Smilax myosotiflora* A.DC.

Kiew RK 1147B

**
STYRACACEAE
**

*Styrax benzoin* Dryand.

Henderson

**
THYMELAEACEAE
**

*Gonystylus maingayi* Hook.*f*.

Henderson

**
TORRICELLIACEAE
**

*Aralidium pinnatifidum* (Jungh. & de Vreise) Miq.

Henderson

**
URTICACEAE
**

*Nothocnide mollissima* (Blume) Chew

Henderson

*Poikilospermum suaveolens* (Blume) Merr.

Henderson

**
VIOLACEAE
**

*Rinorea anguifera* (Lour.) Kuntze

Henderson

*Rinorea horneri* (Korth.) Kuntze

Henderson

**
VITACEAE
**

*Ampelocissus cinnamomea* (Wall.) Planch.

Henderson

*Cayratia mollissima* (Wall.) Gagnep.

Henderson

*Cayratia japonica* (Thunb.) Gagnep.

Kiew RK 223

*Cissus hastata* Miq.

Henderson

*Leea indica* (Burm.*f.*) Merr.

Henderson

**
ZINGIBERACEAE
**

*Camptandra parvula* (King *ex* Baker) Ridl.

Kiew RK 1177, Saw FRI 34267

*Etlingera littoralis* (J.Koenig) Giseke

Henderson

Globba patens Miq. var. costulata S.N.Lim

Kiew RK 1082

Globba pendula Roxb. var. pendula

Henderson

**^E^** Globba variabilis Ridl. var. variabilis

Henderson

*Zingiber gracile* Jack

Kiew RK 1174
